# Transcriptome and Proteome Analysis Identify Decorin as a Principal Antifibrotic Component Trapping TGF-*β*1 Within Adipose-Derived Stem Cell Secretome

**DOI:** 10.1155/sci/1416567

**Published:** 2025-05-09

**Authors:** Lin Kang, Zhujun Li, Fangyuan Li, Ziming Li, Liquan Wang, Tianhao Li, Jieyu Xiang, Songlu Tseng, Nanze Yu, Jiuzuo Huang, Xiao Long

**Affiliations:** ^1^Biomedical Engineering Facility of National Infrastructures for Translational Medicine, Institute of Clinical Medicine, Peking Union Medical College Hospital, Chinese Academy of Medical Sciences and Peking Union Medical College, Beijing 100730, China; ^2^Department of Plastic and Reconstructive Surgery, Peking Union Medical College Hospital, Chinese Academy of Medical Sciences and Peking Union Medical College, Beijing 100730, China

**Keywords:** ADSCs, antifibrosis, decorin, scleroderma, secretome, TGF-*β*

## Abstract

Adipose-derived stem cells (ADSCs) demonstrated therapeutic potential in various fibrotic diseases, with their paracrine proteins playing a crucial role. Nonetheless, the principal paracrine factors of ADSCs responsible for antifibrosis have not yet been well identified. To address this issue, we initially confirmed that ADSCs could attenuate fibrosis and suppress TGF-*β*1 in bleomycin-induced skin fibrosis mouse models. RNA-sequencing of the cocultured fibroblasts demonstrated that ADSCs effectively inhibited the TGF-*β*/Smad2 signaling pathway in fibroblasts through the paracrine approach. Proteomic analysis of the cell supernatant (CS) demonstrated a significant upregulation of 97 proteins in the secretome of ADSCs, among which decorin (DCN) exhibited a particularly elevated level of overexpression. Protein–protein interaction (PPI) network analysis indicated a strong correlation between DCN and TGF-*β*1, with DCN effectively trapping TGF-*β*1 through core protein binding. Cell experiments demonstrated that DCN could effectively inhibit TGF-*β*1-induced fibroblast proliferation. Therefore, it was concluded that DCN was a crucial protein in ADSC secretome that exerted antifibrotic effects by inhibiting TGF-*β*1. This study conducted an in-depth insight into the paracrine function of ADSCs through transcriptome and proteome analysis, identifying DCN as an essential paracrine factor mediating the antifibrotic effect of ADSCs, which could provide valuable theoretical support for the use of ADSC secretions as well as DCN in the treatment of fibrotic diseases.

## 1. Introduction

Fibrosis is a common pathological feature in various diseases. It is defined as increased fibroblast proliferation and deposition of extracellular matrix (ECM) components [1, 2]. Fibrosis involves multiple systems and organs, for example, lung [3], liver [4], kidney [5], skin [6], heart [7], and skeletal muscle [8], with potential clinical ramifications including organ dysfunction and failure. Despite the huge burden of fibrosis-related diseases, there are currently limited specific treatments for fibrosis. Therefore, novel antifibrosis drugs and strategies need to be urgently developed.

Adipose-derived stem cells (ADSCs) belong to the family of mesenchymal stem cells (MSCs) that have self-renewal and multipotent capacities [9, 10]. ADSCs have emerged as a favorable option for adult stem cells owing to their abundant source, ease of collection, and lower immunogenicity [11, 12]. Interestingly, numerous researches have demonstrated that ADSCs are promising antifibrosis agents, with studies demonstrating their ability to inhibit fibrosis in various diseases such as hypertrophic scar [13], keloid [14, 15], and pulmonary fibrosis [16, 17]. Additionally, our previous research demonstrated the efficacy of ADSC-assisted fat grafting in attenuating skin fibrosis and improving fat retention in localized scleroderma (LoS) [18]. Although ADSCs have shown effective antifibrotic capabilities both in vitro and in vivo, the underlying mechanisms of ADSCs to resist fibrosis still require further exploration.

The paracrine factors of stem cells, referred to as secretome, consisting of extracellular vesicles and a variety of proteins, including cytokines, chemokines, and growth factors, have garnered increasing attention in basic research and clinical applications [19, 20]. Recently, more and more evidence suggested that ADSC–based fibrosis treatment could be mediated through paracrine effects. Li et al. [21] reported that the conditioned medium of ADSCs was able to decrease collagen deposition and scar formation so that suppress hypertrophic scar fibrosis. Yang et al. [22] demonstrated that ADSCs inhibited dermal fibroblast growth in keloids through the arachidonic acid-derived cyclooxygenase-2/prostaglandin E2 cascade by paracrine. Therefore, these findings collectively suggest that paracrine secretions of ADSCs contain factors with antifibrotic properties. However, the specific proteins within the secretome of ADSCs that contribute to the antifibrotic effect are not fully understood.

In this study, we initially assessed the therapeutic effect of ADSCs on skin fibrosis in a mouse model induced by bleomycin. On this basis, transcriptome sequencing analysis was conducted on cocultured fibroblasts to explore the signaling pathways involved in the inhibition of fibrosis by ADSC supernatant. Furthermore, to identify the key secretory factors contributing to anti-fibrosis, ADSC secretome was identified through tandem mass tag (TMT)-labeled mass spectrometry (MS). Through a comparison of the differential proteome between the cell supernatant (CS) group of ADSCs and the pure cell medium (CM) group, we identified decorin (DCN) as a key antifibrosis factor using bioinformatics analysis, which was further verified at the cellular level. Herein, this project investigated the antifibrotic properties of ADSCs at the animal, transcriptome, and proteome levels, to determine DCN as a principal paracrine factor of ADSCs in antifibrosis, so that provide crucial theoretical and data support for the potential utilization of ADSC secretome in antifibrosis therapy.

## 2. Materials and Methods

### 2.1. Materials

Trypsin was purchased from Promega. TMT16plex Isobaric Label Reagent Set was purchased from Thermofisher Scientific. Fluorescent-labeled antibodies including CD105, CD29, CD73, et cetera were purchased from BioLegend. ADSC differentiation media for osteoblasts, adipocytes, and chondrocytes were from Cyagen Biosciences. Bleomycin hydrochloride was purchased from MedChemExpress. Four percent paraformaldehyde, H&E staining kit, and Masson's trichrome staining kit were provided by Servicebio. DCN enzyme-linked immunosorbent assay (ELISA) kit was from Solarbio. Recombinant human DCN protein was purchased from Abcam. DCN neutralizing antibody (DCN Ab) was purchased from R&D Systems.

### 2.2. ADSC Culture and Identification

ADSCs were obtained from Fuyuanbio Co., Ltd. (Shanghai, China). The cells were cultured in a specific serum-free complete medium for ADSCs at 37°C in a humidified atmosphere containing 5% CO_2_. The ADSCs were passaged every 3 days and the third passage (P3) was used in the following experiments.

ADSCs characterization was carried out using a flow cytometer. Briefly, 1 × 10^6^ ADSCs in the logarithmic growth phase were collected and incubated with fluorescent-labeled antibodies (CD11b, CD19, CD34, CD45, CD73, CD90, CD105, HLA-DR, and isotype controls). The mixtures were then incubated in the dark in an ice-slurry bath for 30 min, washed twice with ice buffer, and resuspended in 500 μL buffer. Finally, the percentage of cells expressing mesenchymal markers was determined by analyzing the cells using a FACSCalibur flow cytometer (BD Biosciences, USA).

To characterize the differentiation properties of stem cells, ADSCs were cultured in differentiation media for osteoblasts (Cyagen Biosciences., HUXMD-90021), adipocytes (Cyagen Biosciences., HUXMD-90031), or chondrocytes (Cyagen Biosciences., HUXMD-90041), respectively. As manufacturer's protocols: for osteoblasts, ADSCs were cultured with osteogenic differentiation medium for 3 weeks and stained with Alizarin red; for adipocytes, ADSCs were cultured with adipogenic differentiation medium A and medium B in turn until lipid droplets formed and finally stained by Oil red O; for chondrocytes, ADSCs were aggregated into spheroids through centrifugation and cultured with chondrogenic differentiation media for 3 weeks, then fixed and stained with Alcian blue.

### 2.3. Skin Fibrosis Mouse Models and Intervention

Skin fibrosis mouse models were induced with bleomycin subcutaneous injection. The ethical committee of Peking Union Medical College Hospital approved the animal experiments, which were conducted in compliance with the NIH Guide for the Care and Use of Laboratory Animals (XHDW-2021-044). At 6 weeks of age, 18 female BALB/C nude mice were randomly divided into three groups: PBS control, Model control, and ADSC treatment. Each group consisted of six mice. For a period of 30 days, the mice in the model control and ADSC treatment groups were subcutaneously injected with 0.1 mL of 200 μg/mL bleomycin hydrochloride solution in the lower back every day. At the same location, 0.1 mL of PBS was administered to the PBS control group. After successful skin fibrosis model construction, subcutaneous injections of 1 × 10^6^ ADSCs suspended in 0.1 mL PBS were administered to the ADSC treatment group, while the PBS control and model control groups received an equal volume of PBS alone at the same site. After a month, the mice were euthanized and the fibrotic skin was used for further research.

### 2.4. Histological Staining

Mouse skin samples were fixed overnight in 4% paraformaldehyde solution at 4°C and embedded in paraffin. For H&E staining, 5 μm sections were deparaffinized in xylene, rehydrated in alcohol, and rinsed in distilled water. Rehydrated sections were stained with H&E staining kit following the manufacturer's protocol.

Masson staining was performed with the Masson's trichrome stain kit following the manufacturer's protocol. The blue-stained collagen area was quantified by Image J. Collagen volume fraction (CVF) was calculated by the following equation:

  $$\begin{array}{c} \text{CVF }\left(\%\right)=\left(\text{Collagen area}/\text{full field area}\right)\times 100\%. \end{array}$$

For immunohistochemistry staining, the sections were deparaffinized, rehydrated, and rinsed in distilled water. Antigens were retrieved by heating and sodium citrate buffer (pH = 6.0). Endogenous peroxidase was eliminated by 3% H_2_O_2_ treatment. After that, the sections were blocked with 3% bovine serum albumin for 30 min and then incubated in primary antibodies at 4°C overnight. After washing with PBS, sections were incubated with horseradish peroxidase (HRP)-conjugated secondary antibodies, treated with diaminobenzidine (DAB), stained with hematoxylin, dehydrated, and covered with a mounting medium. Skin samples from healthy donors and patients with LoS were selected for immunohistochemistry analysis of DCN, which was approved by the Ethics Committee of Peking Union Medical College Hospital (ZS-2786).

### 2.5. Coculture of ADSCs and Fibroblasts

Fibroblasts were isolated from the skin of patients with LoS and approved by the ethics committee of Peking Union Medical College Hospital. Transwell coculture system with 0.4 μm pore size (3450, Corning, MA, USA) was utilized. ADSCs were cultivated at a density of 1 × 10^4^ cells/cm^2^ in the upper chamber, while fibroblasts were seeded in the below chamber. After 48 h, the fibroblasts in the bottom chamber were collected for bulk RNA sequencing. Fibroblasts without ADSC intervention were used as controls.

### 2.6. Bulk RNA Sequencing

The total RNA of the cocultured fibroblasts in the lower chamber was extracted using the TRIzol reagent, fragmented into short segments, and converted into cDNA. After being ligated with sequencing adapters and amplified, the samples were sequenced by the Illumina Novaseq 6000 platform. The R package limma was used to calculate differentially expressed genes (DEGs). The significance threshold was set at adjusted *p* value (*p*.adjust) < 0.05 and |log2 (fold change)| > 1. The normalized expression matrix was calculated with limma.

### 2.7. ADSC Supernatant Collection

To obtain CS, ADSCs were seeded in T75 flasks at a density of 8000 cells/cm^2^ and incubated for 72 h until they reached 90%–95% confluence. The resulting CS was then collected and transferred to a centrifuge tube. After centrifugation at 2000 × *g* for 10 min at 4°C, dead cells and cell debris were removed. The acquired supernatant was stored at −80°C until protein extraction. At the same time, another equal amount of CM was placed in the incubator for 72 h as the control group. Each group consisted of three independent biological replicates.

### 2.8. Proteomics Analysis of ADSC Secretome

High-abundance proteins in ADSC supernatant were depleted using ProteoMiner Protein Enrichment Kits (Bio-Rad, USA) according to the manufacturer's instructions. The extracted proteins from each sample were subjected to reduction and digested with trypsin. Then the samples were labeled with TMT reagents and fractionated into fractions by high pH reverse-phase high-performance liquid chromatography (HPLC). Subsequently, the peptide fractions were dissolved in 0.1% formic acid and analyzed using the nano UPLC-MS/MS system consisting of a Nanoflow HPLC system (EASY-nLC 1000 system, Thermo Scientific) and Orbitrap Exploris 480 mass spectrometer (Thermo Scientific). At last, the resulting MS/MS data were searched using Proteome Discoverer 2.4 with 15 ppm mass tolerance for precursor ion. The false discovery rate (FDR) was adjusted to <1%. Differentially expressed protein (DEP) was identified as that with over 1.5- or ≤1/1.5-fold change as the cutoff value.

### 2.9. Bioinformatic Analysis

Functional enrichment analysis was carried out using clusterProfiler, with a cutoff value of *p* < 0.05. The Gene Ontology (GO) was used to analyze the biological attributes, such as biological processes (BPs), cellular components (CCs), and molecular functions (MFs) of the DEGs. The Kyoto Encyclopedia of Genes and Genomes (KEGG) database provided information on significantly enriched pathways. *p* value < 0.05 was set as the cutoff value for significant enrichment. The protein–protein interaction (PPI) network of the selected gene was constructed with the STRING database (https://string-db.org). Gene set enrichment analysis (GSEA) was carried out with GSEAbase. Concordantly changed gene sets were considered significantly altered pathways. The FDR *q*-value < 0.05 was set as the threshold for statistical significance. The R package termed “WGCNA” was used to construct the gene co-expression network, perform the hierarchical clustering, and calculate the eigengenes. The correlation between modules and treatments was calculated. The top 1% genes with the highest intramodule connectivity were considered as hub genes.

### 2.10. ELISA

The DCN content in the supernatant of ADSCs was detected by ELISA using a ELISA kit according to the manufacturer's protocol. In brief, CS or CM were added into the microplate coated with monoclonal antibody. After 90 min incubation, biotinylated secondary antibody, HRP-labeled streptavidin and tetramethylbenzidine (TMB) were added in sequence. Finally, optical density of samples was read at wavelengths of 450 and 630 nm using a Microplate Reader (Synergy HT, BioTEK). The concentration of each sample was calculated according to the standard curve.

### 2.11. Cell Proliferation Inhibition Assay

Human fibroblasts were seeded in a 96-well plate at 5000 cells/well and allowed to adhere. After culturing for 24 h, the cells were treated with TGF-*β*1(2 ng/mL), DCN (15, 150 ng/mL), CS, or DCN Ab. Following a 24 h incubation, 10 μL of CCK-8 solution was added to each well and incubated for another 2 h. Finally, the absorbance at 450 nm was quantified using a Microplate Reader.

### 2.12. Statistical Analysis

Samples were conducted at least in triplicate, all the experimental data were expressed as the mean ± SD. Statistical analysis was performed using SPSS Statistics version 21.0. Student's *t*-test was used for comparisons of two groups. A *p* value < 0.05 was considered statistically significant.

## 3. Results

### 3.1. Characteristics of ADSCs

**Figure 1:**
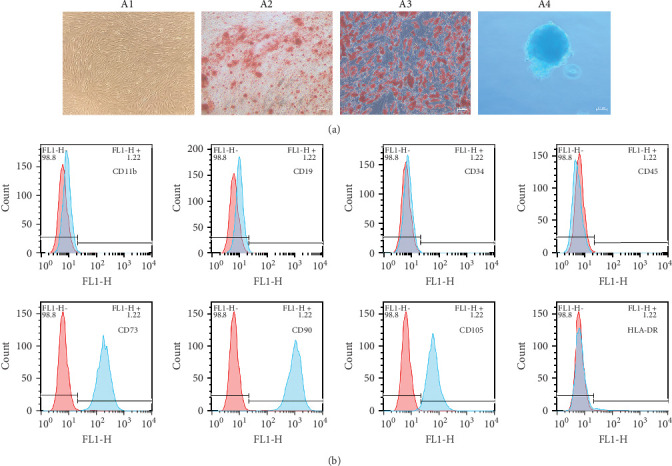
Adipose-derived stem cells (ADSCs) phenotypic evaluation. (A) Representative images of ADSCs (A1); photos of ADSCs cultured in differentiation media and stained by Alizarin red (A2), Oil red O (A3), Alcian blue (A4), respectively, scale bar: 100 μm. (B) Flow cytometry results of ADSCs corresponding to mesenchymal stem cells antibodies, with positive for CD73, CD90, and CD105, and negative for CD11b, CD19, CD34, CD45, and HLA-DR, red plot represent isotype control in each group.

ADSCs are a type of MSCs isolated from adipose tissue and have the general properties of MSCs. The ADSCs used in this research were obtained from Fuyuanbio Co., Ltd. (Shanghai, China) and were subjected to further identification processes to ensure their reliability. The trilineage differentiation experiments showed that ADSCs of P3 were able to be stained with Alizarin red, Oil red O, and Alcian blue after being cultured with osteogenic, lipogenic, and chondrogenic media, respectively (Figure 1A). These results suggested that the ADSCs possessed good multiple differentiation properties. Meanwhile, flow cytometry was employed to evaluate cell surface markers and confirmed that the ADSCs expressed classical MSC phenotype, being positive for CD73, CD90, and CD105 and negative for CD11b, CD19, CD34, CD45, and HLA-DR (Figure 1B). Taken together, these traits were in accordance with the features of MSCs and reinforced their identity as ADSCs, paving the way for the subsequent proteomics analysis.

### 3.2. ADSCs Attenuated Fibrosis and Inhibited TGF-*β* in Mouse Models

**Figure 2:**
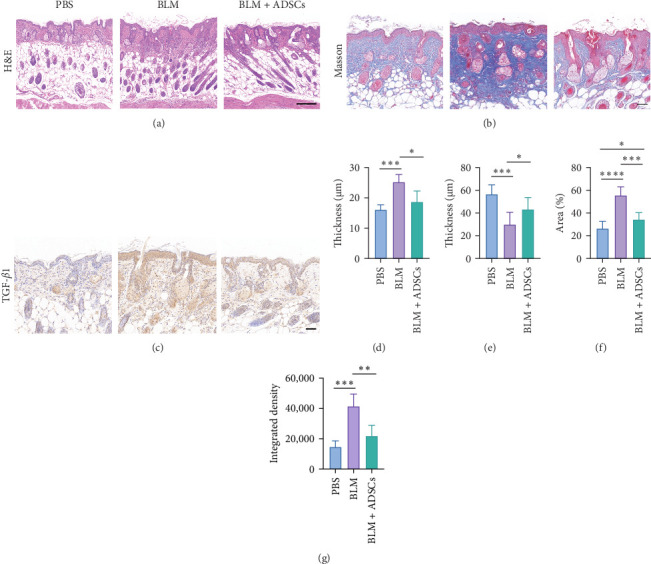
In vivo treatment effects of ADSCs in bleomycin-induced mice models. (A) H&E staining of mice skin (scale bar, 200 μm). (B) Masson's trichrome staining of mice skin (scale bar, 50 μm). (C) The immunochemistry staining of TGF-*β*1 in mice skin (scale bar, 50 μm). (D) Dermal thickness of mice skin. (E) Subcutaneous fat thickness of mice skin. (F) Collagen volume fraction of mice skin in Masson staining. (G) Integrated density of TGF-*β*1 immunochemistry staining. *n* = 6 for each group, *⁣*^*∗*^*p* < 0.05, *⁣*^*∗∗*^*p* < 0.01, *⁣*^*∗∗∗*^*p* < 0.001, *⁣*^*∗∗∗∗*^*p* < 0.0001.

To investigate the antifibrotic effect of ADSCs in vivo, bleomycin-induced skin fibrosis mouse models were established according to the literature [23–25]. 1 × 10^6^ ADSCs were subcutaneously injected into the lesions. Posttreatment, the results of H&E staining revealed thickened dermis and reduced subcutaneous fat after bleomycin injection, whereas the dermal thickness and subcutaneous fat of fibrotic skin were significantly improved after ADSC intervention, as illustrated in Figure 2A,D,E. Masson's trichrome staining was applied to assess the collagen expression in the skin. As a result, the collagen deposition in the Model groups was significantly increased compared to the PBS group and the increased collagen deposition was effectively reversed by ADSC treatment (Figure 2B,F). Further exploration of the fibrosis-related protein TGF-*β* was carried out using immunohistochemistry staining, which demonstrated that the TGF-*β*1 expression levels were significantly upregulated after bleomycin stimulating and ADSCs were able to effectively suppress the expression of TGF-*β*1 (Figure 2C,G), which was thought to be closely related to the alleviation of skin fibrosis.

### 3.3. ADSC Secretions Downregulated the TGF-*β* Signaling in Fibroblasts by RNA-seq

**Figure 3:**
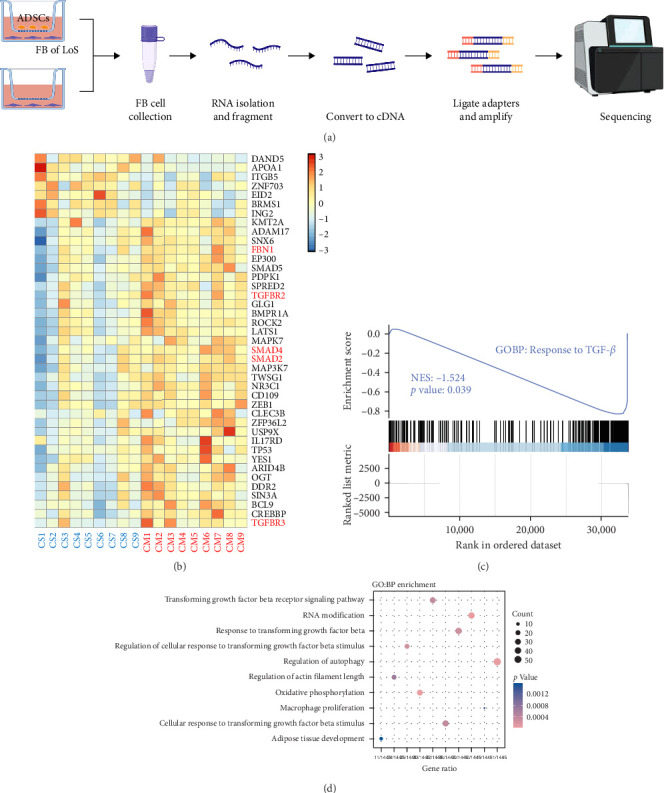
Bulk RNA-sequencing of ADSCs cocultured fibroblasts (FB) extracted from localized scleroderma (LoS) patients. (A) The experimental workflow of the RNA-sequencing. (B) Heatmap of differentially expressed genes between ADSC supernatant (CS) and cell medium (CM) cultured fibroblasts. (C) Gene set enrichment analysis (GSEA) result of TGF-*β* response in ADSC supernatant treated fibroblasts. (D) The result of GO biological process enrichment based on GSEA analysis.

The TGF-*β* signaling pathway was found to be overactivated in skin fibroblasts from scleroderma patients [26–28]. In this study, ADSCs and fibroblasts from LoS were cocultured using a transwell system. Subsequently, the fibroblasts were isolated for bulk RNA sequencing, the workflow was shown in Figure 3A. As a result, DEGs analysis revealed that TGF-*β* signaling pathway related genes, such as smad2, smad4, and TGFBR2, were significantly downregulated in fibroblasts treated with ADSC secretions (Figure 3B and Figure S1). Meanwhile, as displayed in Figure 3D, the most significantly enriched GO term for BP was annotated as TGF-*β* signaling pathway. Moreover, GSEA was performed to further explore the TGF-*β* related terms between two kinds of fibroblasts. It was found that GO of response to TGF-*β* was significantly downregulated in fibroblasts cocultured with ADSCs (Figure 3C). Additionally, the expression levels of fibrosis-associated mRNAs were quantified according to the normalized expression matrix. The mRNA expression levels, including FN1, ACTA2, COL1A1, COL1A2, and COL3A1, were significantly decreased in ADSC supernatant cocultured fibroblasts, with *p* value < 0.05 (Figure S2). Overall, the results of RNA-seq suggested that the TGF-*β* signaling pathway in scleroderma fibroblasts was significantly downregulated with the intervention of ADSC secretions, leading to the inhibition of the fibrosis-related genes.

### 3.4. WGCNA Analysis Suggested Smad2 as a Hub Gene in Fibrosis Inhibition

**Figure 4:**
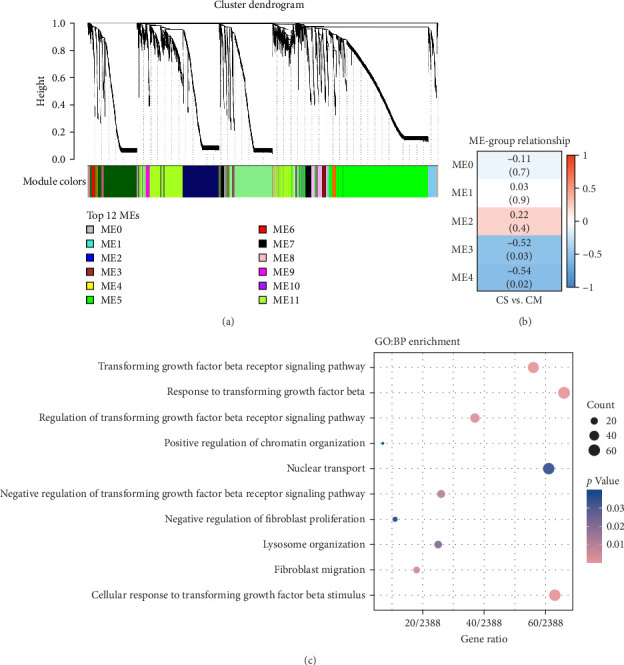
Identification of modules associated with TGF-*β* through weighted gene co-expression network analysis (WGCNA). (A) Clustering dendrogram of WGCNA analysis. (B) Gene modules obtained by WGCNA, ME3 block was selected for subsequent analysis. (C) GO enrichment analysis of biological process for ME3 block.

To understand the gene expression program changes in scleroderma fibroblasts treated with ADSC supernatant, we conducted unbiased WGCNA on the complete dataset. First, sample clustering was performed using Person's correlation coefficient, resulting in the sample clustering tree (Figure S3). The scale-free index and mean connectivity for different soft-threshold powers in WGCNA were illustrated in Figure S4. Next, the cluster dendrogram exhibited the co-expression gene modules identified via WGCNA, as displayed in Figure 4A. Correlation analysis between modules and treatments highlighted ME3 as significantly negatively correlated (*p* < 0.05; Figure 4B). Therefore, ME3 block was selected for subsequent analysis. GO enrichment analysis of BP for ME3 revealed top terms related to TGF-*β* and fibroblast functions, including TGF-*β* signaling pathway, response to TGF-*β*, regulation of TGF-*β* receptor signaling pathway, negative regulation of TGF-*β* receptor signaling pathway, and negative regulation of fibroblast proliferation (Figure 4C). Moreover, hub genes were identified based on the top 1% of genes with the highest intramodule connectivity. As listed in Table S1, the top five genes related to TGF-*β* signaling in Module 3 were ranked by betweenness, Smad2 was recognized as a hub gene in Module 3 due to its association with the TGF-*β* signaling pathway. This analysis supported the hypothesis that ADSCs might secrete active components to inhibit TGF-*β*, subsequently downregulating Smads and its downstream COL1, COL3, et cetera, ultimately leading to fibrosis attenuation in fibroblasts.

### 3.5. Proteomic Analysis Revealed a Significant Increase of DCN in ADSC Supernatant

**Figure 5:**
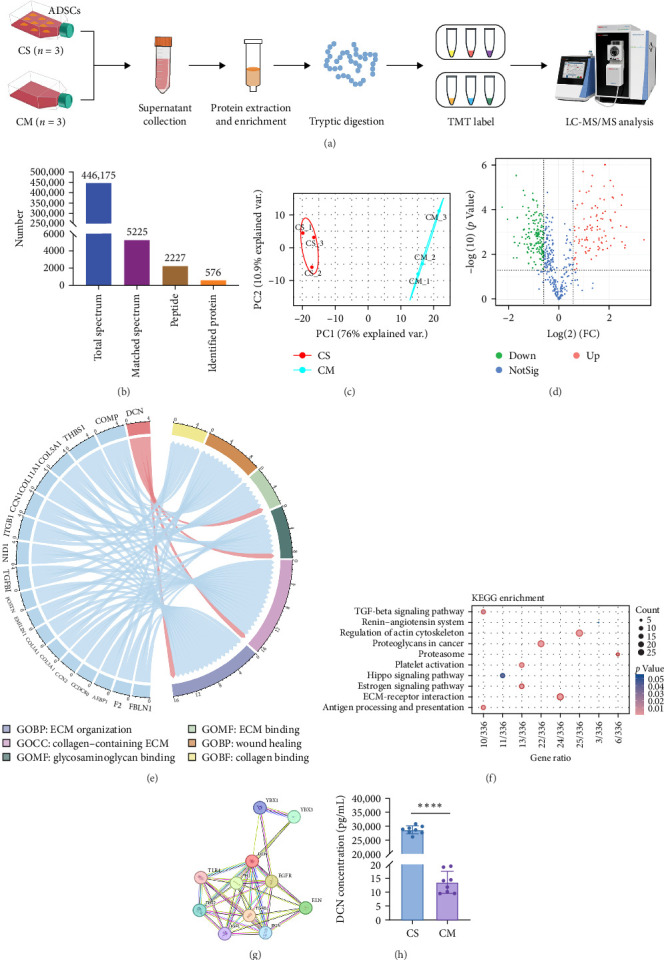
TMT–based quantification of ADSCs secretome. (A) Workflow of the proteomic analysis, cell supernatant (CS), and cell medium (CM). (B) Protein identification results, 576 proteins (unique peptides ≥1) were identified from 2227 unique peptides. (C) PCA analysis of CS and CM. (D) Volcano plot of differentially expressed proteins between CS and CM. (E) Chord diagram for GO enrichment of biological process (BP), molecular function (MF), and cellular component (CC). (F) KEGG pathway enrichment results of upregulated proteins in ADSCs supernatant. (G) Protein–protein interaction (PPI) network of decorin (DCN) according to STRING database. (H) The content of DCN in ADSCs supernatant and cell media detected by ELISA, *n* = 8.

The supernatant of ADSCs was collected for proteomic assay and referred to as the CS group, while the pure CM incubated under the same condition served as the control group and was referred to as the CM group. Both CM and CS samples were digested with trypsin, labeled with TMT, and subjected to LC-MS/MS analysis, as illustrated in Figure 5A. As a result, a total of 446,175 spectra corresponding to 2227 peptides were obtained. Totally, 576 proteins (with unique peptides ≥1) were identified (Figure 5B). Principal component analysis (PCA) indicated distinct clustering of CS vs. CM samples (Figure 5C). Moreover, proteins with fold change >1.5 and *p* value < 0.05 were considered differentially expressed. A total of 253 proteins were found to be differentially expressed between the two groups, as shown in Figures S5 and S6. In comparison to the CM, 97 upregulated proteins and 156 downregulated proteins were found in the CS (Figure 5D), with the top five upregulated proteins ranked by fold change listed in Table 1.

To comprehend the biological relevance of proteins secreted by ADSCs, we initiated GO enrichment analysis to categorize based on BP, CC, and MF. The top 10 terms in each category were depicted in Figure S7. The chord diagram in Figure 5E represented composite GO terms, highlighting significantly changed proteins in BP, CC, and MF. Particularly, DCN was linked to ECM organization, ECM binding, and collagen-containing ECM, suggesting its crucial role in ECM organization. Furthermore, PPI analysis revealed interactions of DCN with various proteins, including TGF-*β*1 (Figure 5G). Meanwhile, KEGG enrichment analysis demonstrated that the TGF-*β* signaling pathway was significantly changed (Figure 5F). On the other hand, ELISA was employed to quantify the concentration of DCN in supernatant of ADSCs. As a result, the concentration of DCN in CS was significantly higher than that in CM, with levels of 28.65 ng/mL and 13.50 pg/mL, respectively (Figure 5H). This result aligned with the outcomes of the proteomic analysis. These findings demonstrated that DCN was significantly highly expressed in ADSC secretome and DCN might interact with TGF-*β*1 to regulate the TGF-*β* signaling pathway, thereby affecting ECM organization.

### 3.6. DCN Maintained Normal Skin Status and Inhibited TGF-*β* Induced Fibroblast Proliferation

**Figure 6:**
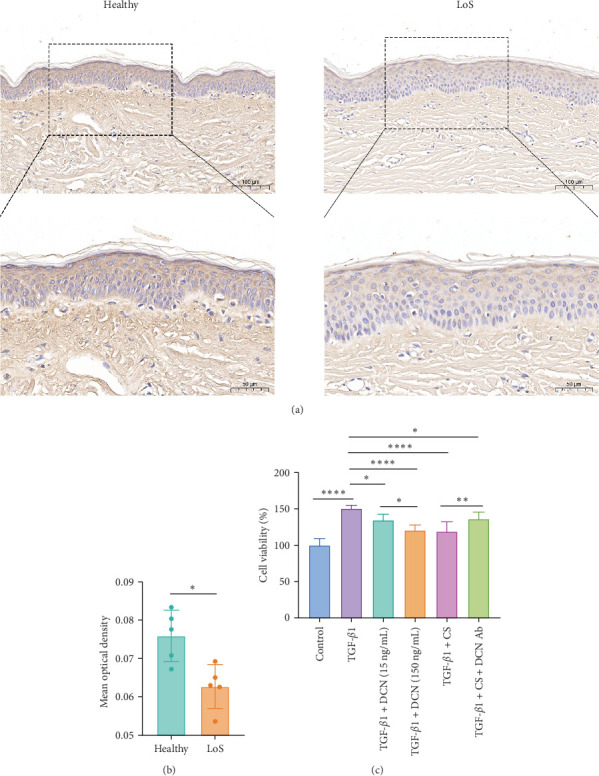
Decorin (DCN) maintained normal skin status and inhibited TGF-*β* induced fibroblast proliferation. (A) Immunochemistry staining for the expression of DCN in skin tissue from healthy individuals and patients with localized scleroderma (LoS), scale bar: 100 μm (up) and 50 μm (down). (B) Statistic analysis of DCN immunochemistry staining, *n* = 5. (C) Proliferation inhibition assay of TGF-*β*1 activated fibroblasts treated with DCN, ADSC supernatant (CS), or DCN neutralizing antibody (DCN Ab), *n* = 8. *⁣*^*∗*^*p* < 0.05, *⁣*^*∗∗*^*p* < 0.01, *⁣*^*∗∗∗∗*^*p* < 0.0001.

DCN has been shown to affect the structure and function of the ECM. In this study, skin samples from healthy donors and patients with LoS were selected for immunohistochemistry analysis of DCN. As a result, the expression level of DCN in LoS was significantly downregulated compared with the normal skin (Figure 6A,B), suggesting that DCN played an important role in maintaining skin homeostasis, with its depletion potentially contributing to the fibrotic process.

Furthermore, a fibroblast proliferation assay was employed to validate the capacity of DCN to inhibit fibrosis. Given that scleroderma fibroblasts express high levels of various cytokines, including TGF-*β*, platelet-derived growth factor (PDGF), endothelin-1, et cetera, these cytokines promote fibroblast proliferation through independent or interactive signaling pathways [29]. Therefore, to specifically examine the inhibitory effect of DCN on TGF-*β*, we utilized normal fibroblasts activated with TGF-*β*1 as baseline samples. As illustrated in Figure 6C, the cell proliferation inhibition assay revealed that DCN effectively suppressed the proliferation of fibroblasts stimulated by TGF-*β*1 and the cell inhibition rate at high concentrations of DCN was significantly increased than that at low concentrations of DCN (*⁣*^*∗*^*p* < 0.05). Furthermore, the CS of ADSCs could significantly inhibit the proliferation of TGF-*β*1 activated fibroblasts (*⁣*^*∗∗∗∗*^*p* < 0.0001), whereas the inhibition was significantly mitigated by adding a DCN Ab (*⁣*^*∗∗*^*p* < 0.01). These findings demonstrated that DCN represented a major antifibrotic factor within the paracrine proteins of ADSCs and DCN could restrain fibroblast hyperproliferation by inhibiting the TGF-*β* signaling pathway.

## 4. Discussion

This study used transcriptome combined with proteome to identify the key proteins in ADSC secreted factors that inhibit fibrosis. In order to investigate the potential of paracrine factors secreted by ADSCs in attenuating fibrosis, fibroblasts from LoS patients were cocultured with ADSCs and subjected to transcriptome sequencing. Bioinformatics analysis of the transcriptome data revealed a significant inhibition of the TGF-*β* signaling pathway in fibroblasts by the ADSC secretions (Figure 3B–D). Furthermore, to determine the principal proteins in the paracrine factors of ADSCs that suppress TGF-*β*, a proteomic analysis of the ADSC secretome was performed. A total of 97 proteins were found to be significantly upregulated in the ADSC supernatant compared to the pure culture medium (Figure 5D). These proteins were notably enriched in the TGF-*β* signaling pathway (Figure 5F), with DCN exhibiting a particularly high level of overexpression (28.65 ng/mL in CS vs. 13.50 pg/mL in CM) and demonstrating a close association with ECM formation (Figure 5E,H). Importantly, PPI network analysis revealed a strong association between DCN and TGF-*β*1 (Figure 5G). Additionally, cell experiments validated the ability of DCN to suppress TGF-*β*1 induced fibroblast proliferation (Figure 6C), suggesting that DCN was a key factor in the ADSC supernatant to exert antifibrotic effects.

It has been demonstrated that overexpression of TGF-*β* leads to fibrosis, whereas the administration of TGF-*β* neutralizing antibodies or inhibitors of the TGF-*β* signaling pathway can prevent fibrosis [30–32]. Animal studies and RNA-sequencing found that ADSCs could significantly reduce the TGF-*β* levels in fibrotic skin and fibroblasts (Figures 2C,G and 3B–D), indicating that ADSCs were able to alleviate fibrosis by inhibiting TGF-*β*. TGF-*β* canonical signaling via Smads has a central role in the development of fibrosis [33, 34]. TGF-*β*1 binds to the TGF-*β* receptor to activate Smad2, leading to the transcription of profibrotic genes and subsequent fibroblast activation and ECM deposition [31, 35–37]. WGCNA analysis identified Smad2 as a hub gene in the treatment of scleroderma fibroblasts by ADSC supernatant (Figure 4A–C and Table S1), suggesting that the paracrine factors of ADSCs exerted antifibrotic effects by inhibiting the TGF-*β*1/Smad2 pathway. Moreover, Meng et al. [38] reported that adiponectin-modified bone marrow-derived MCSs (BMSCs) had the potential to mitigate heart fibrosis through the inhibition of the TGF-*β*1/Smad pathway. In a separate study, Xu et al. [39] demonstrated that MSCs could reverse the dedifferentiation of myofibroblasts into fibroblasts by inhibiting the TGF-*β*/Smad2/3 pathway. Our previous research also found that exosomes derived from ADSCs could attenuate LoS fibrosis by targeting the TGF-*β*/Smad axis [40], highlighting the significance of TGF-*β*/Smad signaling pathway inhibition as a key mechanism for ADSC–based antifibrosis treatment.

DCN, a small leucine-rich proteoglycan found in the ECM, is composed of a core protein and a single glycosaminoglycan chain [41, 42]. It plays a crucial role in various cellular processes such as matrix assembly, fibrillogenesis, and the regulation of cell proliferation [43–45]. Most importantly, DCN is involved in modulating ECM degradation and acts as a protective factor against fibrogenesis. Through binding to TGF-*β*1 via its core protein, DCN effectively traps TGF-*β*1 within the ECM, preventing its binding to cell receptors and acting as an endogenous inhibitor of TGF-*β*1 [46, 47]. In patients with LoS, the skin's DCN levels were significantly lower compared to that of healthy individuals (Figure 6A,B), which could be attributed to the overexpression of TGF-*β*1 during fibrosis, leading to excessive consumption of DCN in the matrix. This observation also suggested that supplementing with DCN could be beneficial in alleviating fibrosis symptoms. In fact, previous studies have demonstrated the therapeutic effects of DCN on various fibrotic conditions, such as pulmonary fibrosis [48, 49], liver fibrosis [50], and epidural fibrosis [51, 52]. Furthermore, DCN gene therapy has been reported as a potential strategy for various fibrotic diseases. For example, Lee et al. [53] demonstrated that DCN-expressing adenovirus decreased collagen synthesis and upregulated matrix metalloproteinases (MMPs) expression in keloid fibroblasts. Similarly, DCN gene therapy has shown potential in preventing fibrosis in kidney [54] and corneal tissues [55]. Additionally, Kwan et al. [56] found miR-181b downregulated DCN in dermal fibroblasts and blocking miR-181b with an antagomiR could increase DCN levels and prevent hypertrophic scarring. These results highlight the promise of DCN as a therapeutic agent for a range of fibrotic diseases.

**Figure 7:**
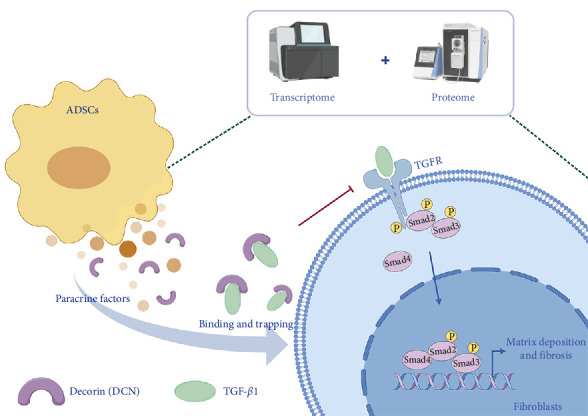
Graphical abstract of this work. Decorin (DCN) was identified as the principal antifibrotic component within the ADSC secretome by combining transcriptomic and proteomic analyses. DCN bound with TGF-*β*1 and prevented it from binding to the TGF receptor, thereby blocking the downstream TGF-*β*/smad2 signaling pathway, ultimately reducing matrix deposition in fibroblasts and alleviating fibrosis.

This study provided new insights by integrating transcriptomic and proteomic approaches to identify DCN as a principal antifibrotic factor in ADSC secretome, which was further validated in vitro (Figure 6C). Although previous investigations have revealed multiple signaling pathways involved in the antifibrotic activity of ADSC secretome, our work specifically highlighted DCN as a crucial functional component (Figure 7). For example, Frommer et al. [57] analyzed publicly available single-cell RNA sequencing data, illustrating the multifunctional role of ADSC secretome in scleroderma treatment. Zhu et al. [58] demonstrated that apoptosis might be one approach by which ADSCs release therapeutic paracrine factors. In a separate study, Oki et al. [59] noted the presence of DCN in the conditioned medium of ADSCs. However, they provided neither methodological details for its identification nor functional validation of its antifibrotic role [59]. Consequently, this work represents a significant advance by validating DCN as a principal antifibrotic factor within the ADSC secretome, elucidating its mechanistic role, and suggesting its potential as a biomarker for evaluating the antifibrotic efficacy of ADSCs.

The cell proliferation inhibition assay indicated that DCN Ab could significantly but not completely eliminate the inhibitory effect of CS (Figure 6C), indicating that there may be other factors inhibiting TGF-*β*1 within the paracrine factors of ADSCs. Given the complexity of stem cell secretions, the present study has only validated the principal component, DCN, without assessing the antifibrotic effects of other upregulated proteins. Meanwhile, this study primarily focused on the role of DCN in inhibiting TGF-*β* and alleviating fibrosis, without investigating the expression of DCN in fibroblasts of scleroderma patients. To address these limitations, follow-up researches could proceed in two directions. On the one hand, the highly expressed proteins in ADSC secretions should be systematically validated to construct a comprehensive antifibrosis protein map of the ADSCs secretome, thereby elucidating the antifibrosis signaling pathway network of ADSCs through paracrine action. On the other hand, the expression of DCN in fibroblasts from scleroderma and other fibrotic diseases should be studied to clarify the mechanism of DCN in fibrosis development and to evaluate the antifibrotic efficacy of DCN–based therapies, including DCN-engineered ADSCs, DCN and their analogs, as well as DCN gene therapy.

## 5. Conclusions

ADSCs demonstrated efficacy in ameliorating bleomycin-induced skin fibrosis, suppressing the expression of TGF-*β*1 in the skin. Transcriptomic analysis revealed a notable inhibition of the TGF-*β* signaling pathway in the fibroblasts of scleroderma patients following exposure to ADSC secretions. Additionally, paracrine factors of ADSCs regulated fibrosis through the TGF-*β*/Smad2 pathway. Furthermore, proteomics identified DCN as a prominently expressed protein in the supernatant of ADSCs. DCN inhibited the TGF-*β* signaling pathway by interacting with TGF-*β*1, as confirmed by in vitro experiments demonstrating its ability to suppress fibrogenesis induced by TGF-*β*1. Ultimately, ADSC supernatant was able to reduce fibrosis by inhibiting the TGF-*β* signaling pathway and DCN was a principal paracrine factor in ADSC supernatant that exerted antifibrosis effects.

## Figures and Tables

**Table 1:** Top five upregulated proteins ranked by fold change.

Gene name	Intensity (CS)	Intensity (CM)	FC (CS_CM)	*p* Value
BGN	1131.26667	107.566667	10.516889	0.002175
DKK1	680.733333	105	6.483175	0.000601
TNFRSF12A	180.166667	28.7666667	6.263036	0.003862
DCN	3168.23333	509.3	6.220761	0.005006
FSTL1	2278.63333	396.433333	5.747835	0.000022

Abbreviations: BGN, biglycan; CM, culture medium; CS, cell supernatant of ADSCs; DCN, decorin; DKK1, dickkopf-related protein 1; FC, fold change; FSTL1, follistatin-related protein 1; TNFRSF12A, tumor necrosis factor receptor superfamily member 12A.

## Data Availability

The data that support the findings of this study are available from the corresponding author upon reasonable request.

## Supporting Information

Supporting Information The supporting images and table of transcriptome and proteome results mentioned in the text can be found here, including Figures S1–S7 and Table S1.
