# Supplementary material for: Transcriptome and Proteome Analysis Identify Decorin as a Principal Antifibrotic Component Trapping TGF-β1 Within Adipose-Derived Stem Cell Secretome
Source: Stem Cells Int. 2025 May 9;2025:1416567. doi: 10.1155/sci/1416567 (PMC12084782; doi:10.1155/sci/1416567)
Supplement: Supporting Information — The supporting images and table of transcriptome and proteome results mentioned in the text can be found here, including Figures S1–S7 and Table S1. [file 1416567.f1.docx]

Figure S1. Volcano plot of differentially expressed genes in RNA sequening.


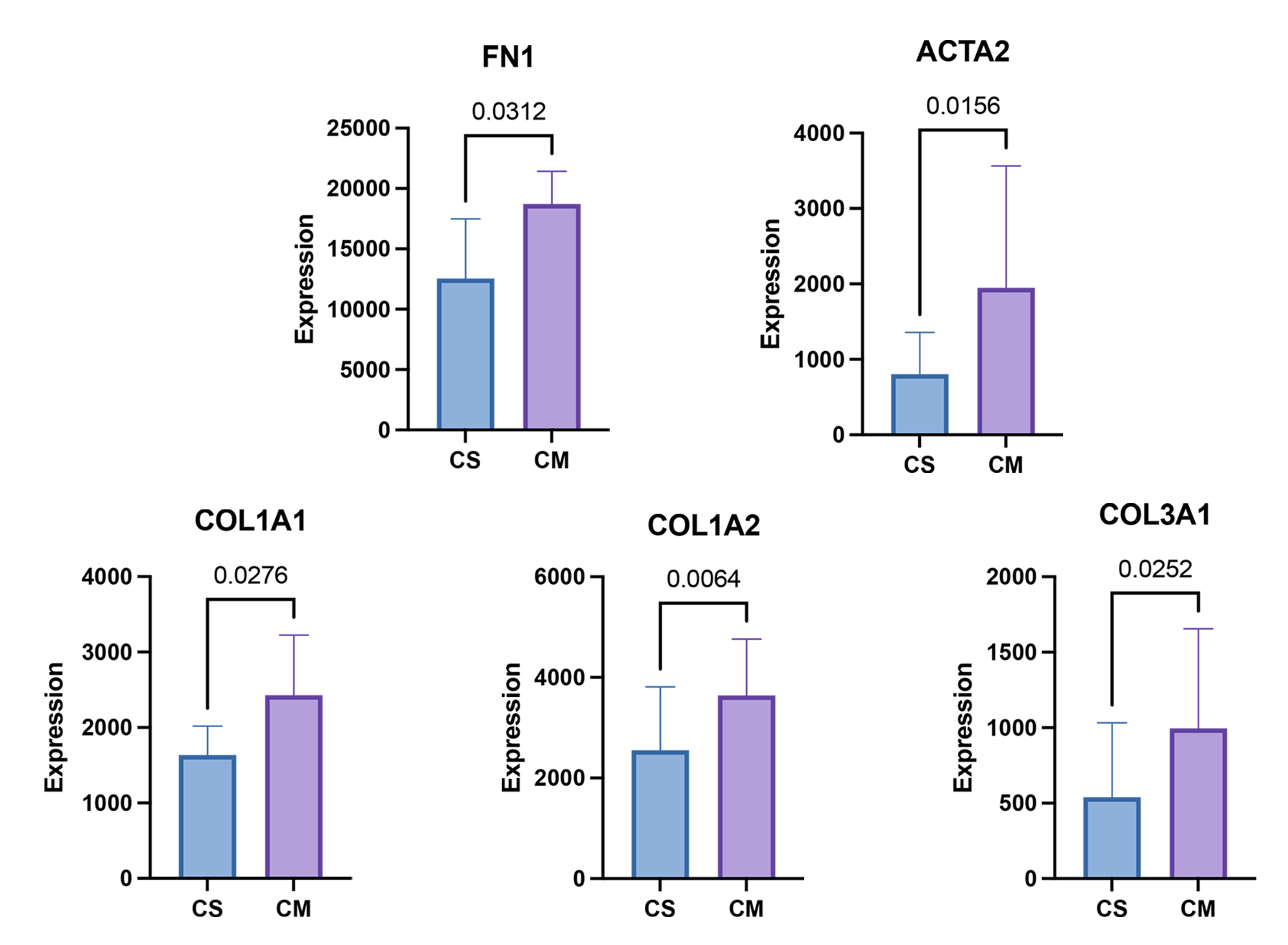


Figure S2. The mRNA expression levels of fibroblasts treated with cell supernatant (CS) or cell culture media (CM).

Figure S3. Clustering dendrogram of samples.

Figure S4. Determination of soft-threshold power in the WGCNA.


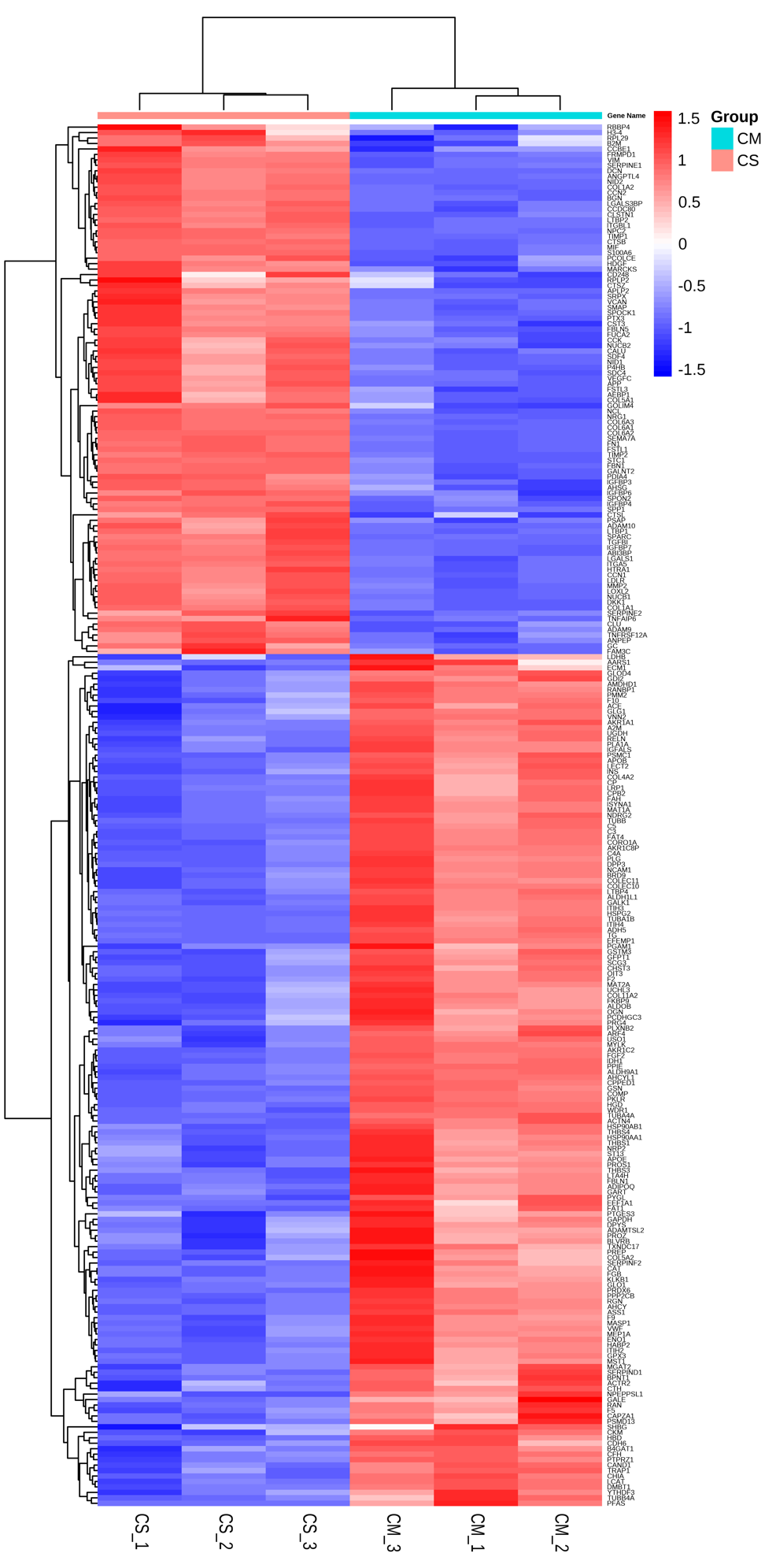


Figure S5. Heatmap based on hierarchical clustering analysis of 253 common differentially expressed proteins between cell supernatant (CS) and cell medium (CM).


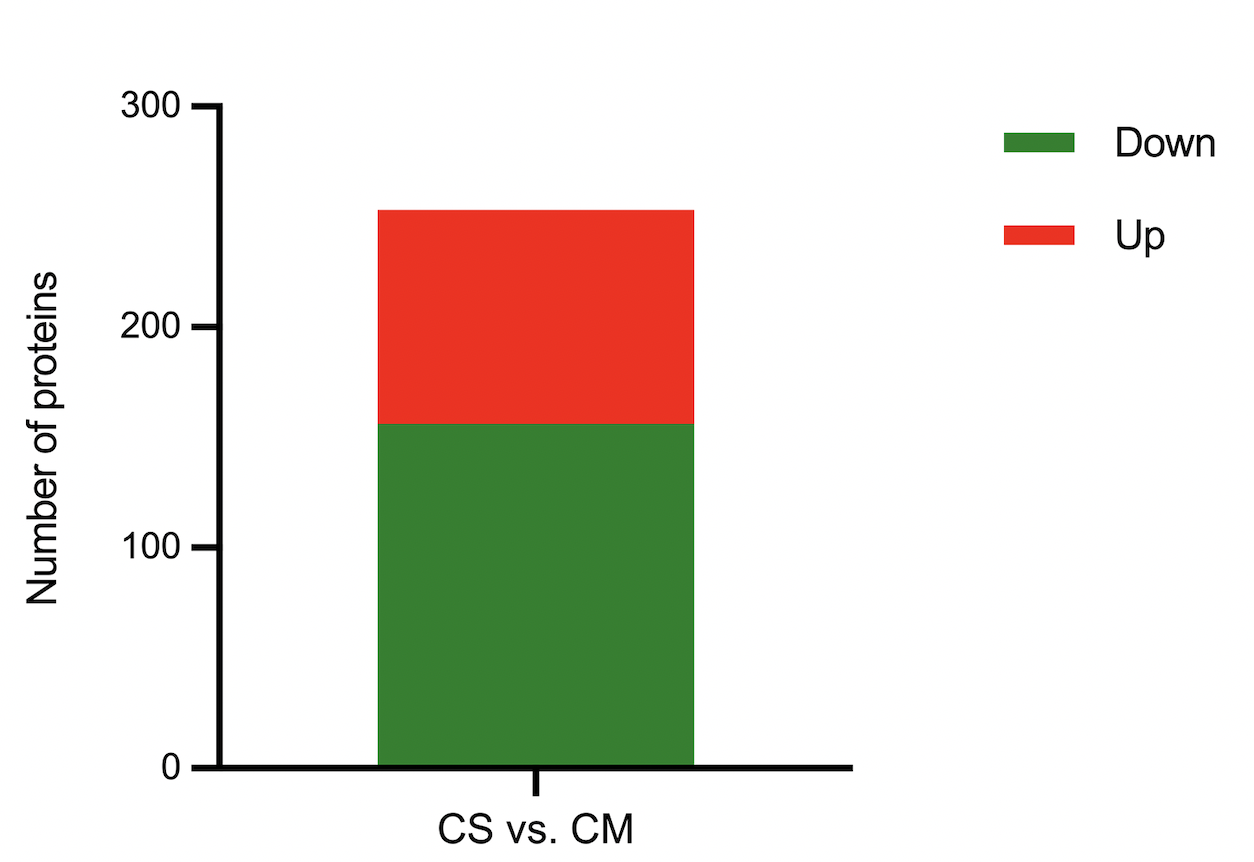


Figure S6. The number of significantly upregulated and downregulated proteins. Compared with cell media, 97 upregulated proteins and 156 downregulated proteins were identified in the cell supernatant of ADSCs (fold change > 1.5 and P value < 0.05).


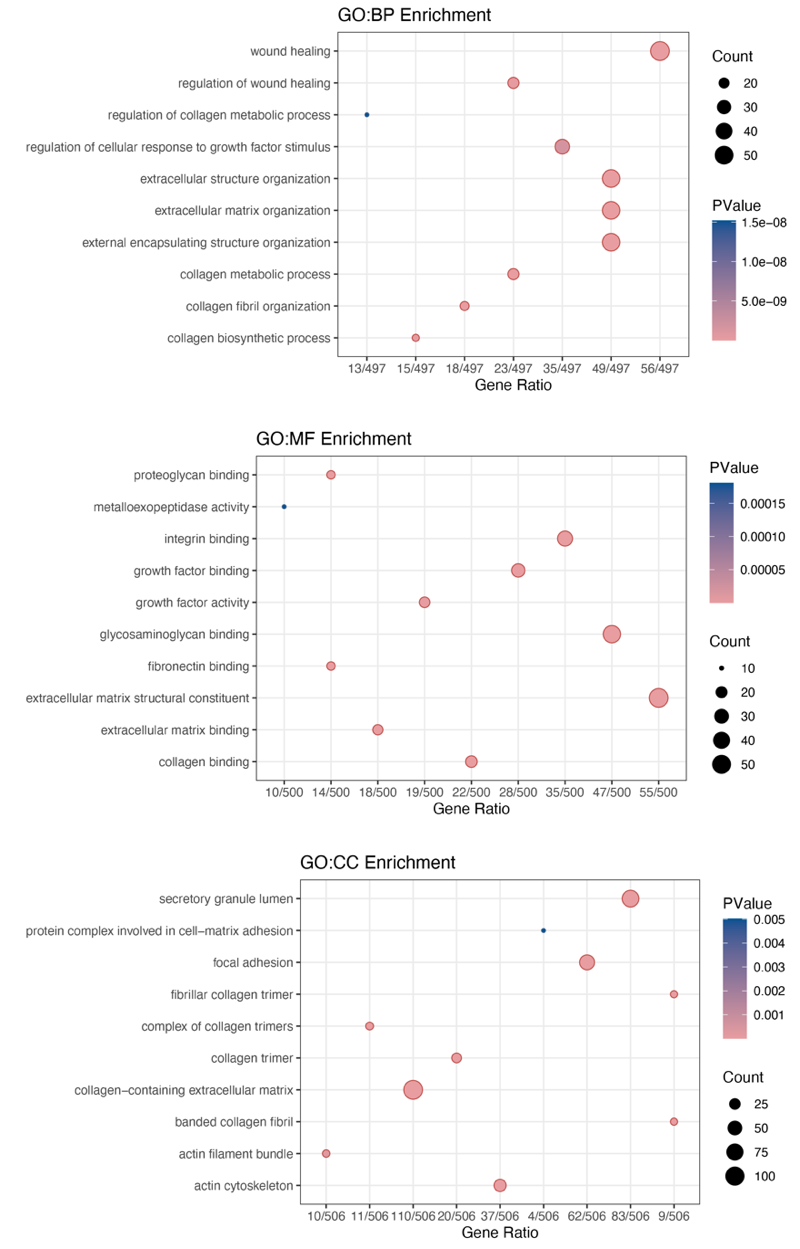


Figure S7. Gene ontology (GO) enrichment analysis of differentially expressed proteins.

Table S1. Top 5 genes related to TGFβ signaling in module 3 ranked by betweenness.

| Gene Name | Betweenness | Degree | Closeness | MCC |
| --- | --- | --- | --- | --- |
| SMAD2 | 22915.98278 | 249 | 1610.41667 | 5710 |
| LATS1 | 19120.7084 | 261 | 1620 | 6991 |
| SUDS3 | 18773.01041 | 226 | 1590 | 4504 |
| ROCK2 | 17406.82353 | 223 | 1599.58333 | 4866 |
| MAP3K7 | 16803.65776 | 244 | 1608.08333 | 7136 |

SMAD2, SMAD Family Member 2. LATS1, Large Tumor Suppressor Kinase 1. SUDS3, SIN3A Corepressor Complex Component. ROCK2, Rho Associated Coiled-Coil Containing Protein Kinase 2. MAP3K7, Mitogen-Activated Protein Kinase Kinase Kinase 7. MCC, Maximal Clique Centrality.
